# 4-(2-Butyl-6,7-dichloro-2-cyclopentyl-indan-1-on-5-yl) oxobutyric acid inhibits angiogenesis *via* modulation of vascular endothelial growth factor receptor 2 signaling pathway

**DOI:** 10.3389/fcvm.2022.969616

**Published:** 2022-09-23

**Authors:** Tianli Zhou, Yunda Li, Heqiang Zhang, Lei Pan, Jinglong Pang, Qian Yuan, Guiyang Li, Lingjun Jie, Yan Wang, Yanhui Zhang

**Affiliations:** ^1^Institute of Cardiovascular Diseases, Xiamen Cardiovascular Hospital of Xiamen University, School of Medicine, Xiamen University, Xiamen, China; ^2^Department of Cardiology, Xiamen Cardiovascular Hospital of Xiamen University, School of Medicine, Xiamen University, Xiamen, China

**Keywords:** 4-(2-Butyl-6, 7-dichloro-2-cyclopentyl-indan-1-on-5-yl) oxobutyric acid angiogenesis, angiogenesis, endothelial cell, vascular endothelial growth factor receptor 2, volume-regulated anion channel

## Abstract

4-(2-Butyl-6,7-dichloro-2-cyclopentyl-indan-1-on-5-yl) oxobutyric acid (DCPIB), was discovered to be a potent and specific antagonist of volume-regulated anion channel that is closely linked to angiogenesis. However, the effect of DCPIB on angiogenesis remains unclear. Here, we found that DCPIB inhibited angiogenesis in the corneal suture and myocardial infarction *in vivo* model. In addition, DCPIB inhibited human umbilical vein endothelial cell migration, tube formation and proliferation *in vitro*. Moreover, DCPIB repressed the activation and expression of vascular endothelial growth factor receptor 2 (VEGFR2) and its downstream signaling pathway. Computer modeling further confirmed that DCPIB binds with high affinity to VEGFR2. Collectively, we present evidence supporting an antiangiogenic role of DCPIB by targeting VEGFR2 signaling pathway, which suggests that DCPIB is a valuable lead compound for the treatment of angiogenesis-related diseases.

## Introduction

Angiogenesis, the formation of new capillaries from pre-existing vascular networks, is comprised of a series of cellular events. Angiogenesis is a sophisticated multi-step process involving five distinct sequential events: 1) angiogenic factors increased endothelial cells (ECs) permeability and proliferation; 2) degradation of basement membrane by activated matrix metalloproteinases; 3) ECs migrate into the interstitial space and sprout and proliferate; 4) Integrins in ECs contribute to lumen formation, elongation and remodeling; 5) Pericytes recruitment, vessel stabilization and finally blood flow. Angiogenesis plays a critical role in many physiological and pathological processes, including embryonic development, the female reproductive cycle, wound healing, myocardial infarction, cancer growth, corneal injury and chronic inflammation (1, 2). Abnormal angiogenesis and deficient angiogenesis both facilitate numerous diseases. Corneal neovascularization characterized by angiogenesis in the normally avascular cornea is a sight-threatening disease. Pathological angiogenesis in tumor accelerates tumor growth. On the contrary, proangiogenesis can provide therapeutic benefits in ischemic diseases, such as ischemic heart disease and ischemic stroke.

In recent decades, a variety of signaling including VEGF–VEGF receptors, angiopoietin-Tie, ephrin-Eph receptors and Notch system, have been implicated in the regulation of angiogenesis. Of them, VEGF is a key mediator of angiogenesis and exerts its pro-angiogenic effects by binding to VEGFR2 on the ECs (3). VEGF-induced VEGFR2 dimerization and autophosphorylation activate downstream signaling cascades including AKT, STAT3, ERK, RhoA and CyclinD1 required for ECs proliferation, migration and tube formation (4–6). Therefore, VEGF/VEGFR2 signaling pathway holds the potential to be an attractive target for pro-and anti-angiogenesis therapy. Macugen and Lucentis are used to treat ophthalmic diseases *via* anti-VEGF therapy. Bevacizumab, sorafenib and sunitinib interfere with tumor angiogenesis by preventing the VEGF–VEGFR signaling pathway. In addition, therapeutic angiogenesis can be used in ischemic cardiovascular disease, such as myocardial infarction. Unfortunately, multiple trials have been performed and currently there are no angiogenic drugs proved by FDA to treat ischemic heart disease.

DCPIB was originally identified as a selective antagonist of volume-regulated anion channel (VRAC) (7). VRAC is expressed widely in mammalian cells and is essential for cell volume homeostasis (8). Subsequent work revealed its role in the regulation of cellular homeostasis and function, including cell differentiation, proliferation, survival, migration and intercellular communication (9–11). VRAC is closely linked with angiogenesis. VRAC blockers including flavonoids, mibefradil, tamoxifen, clomiphene and NPPB inhibited ECs proliferation and angiogenesis *in vivo* and *vitro* (12–14). Selective inactivation of endothelial LRRC8A, the key component of VRAC, resulted in impaired angiogenesis (15). DCPIB was shown, paradoxically in another study, to increase angiogenesis and enhance blood flow recovery in the hind limb ischemia model (16). A recent report provided evidence that the low-dose DCPIB increased the protein expression of the molecular determinant of VRAC (17), which may account for the proangiogenic effect of DCPIB. To date, the role of DCPIB in angiogenesis remains to be further elucidated.

Here, we investigated the potential role of DCPIB in angiogenesis. Our results demonstrated that DCPIB reduced angiogenesis both *in vivo* models of corneal suture (CS) and myocardial infarction (MI). In addition, DCPIB also repressed human umbilical vein endothelial cells (HUVECs) proliferation, tube formation and migration by suppressing the total and phosphorylated VEGFR2 level and its downstream signaling pathway. Moreover, computer modeling also showed that VEGFR2 is the potential molecular target of DCPIB. Taken together, these results indicate that DCPIB has the potential to become a therapeutic agent in the treatment of angiogenesis-related diseases.

## Materials and methods

### Animals and treatments

All animal experiments were reviewed and approved by the Animal Care Committee of Xiamen Univerisity. C57BL/6J mice (6–8 weeks) from Xiamen University Animal Center (Fujian, China) were used in the study. The animals were housed at a density of 5 mice per cage under the conditions of controlled relative humidity of 55–65% and temperature of 23–25°C. Mice CS model was performed as previously described (18). Mice anesthetized and then sutured with 11-0 nylon sutures into the peripheral corneal stroma. Eye drop administration and subconjunctival injection of DCPIB (eye drop: 300 μM; subconjunctival injection: 10 μM) were used in mice (*n* = 5~6 per group). The surgical MI model was induced by permanent ligation of the left anterior descending coronary branch (19). Mice were intraperitoneally injected with DCPIB (15 mg/kg/days) for 2 weeks. After all animals are euthanized, blood, heart tissue and corneal tissue were collected to measure angiogenesis markers and liver enzymes, i.e., alanine aminotransferase (ALT) and aspartate aminotransferase (AST).

### Corneal neovascularization phenotype analysis

Slit-lamp photography (Micron2, Phoenix Research Laboratories, Pleasanton, USA) was used for collecting morphological data and assessing corneal neovascularization by an experienced ophthalmologist on days 0, 3, 5, 7, 10, and 14 after the procedure. Tropicamide was used for pupil dilation. The corneal neovascular area was assessed as described previously (20). Scoring was carried out according to the rule described previously and the average score per group was then obtained (2).

### Cardiac echocardiography

The cardiac function was observed by echocardiography in mice treated with MI procedure (30 MHz transducer, Vevo 2100, VisualSonic Inc., Canada). In brief, isoflurane was used to anesthetize mice. M-mode and two-dimensional echocardiography were performed to assess cardiac systolic function in mice.

### Cell culture

HUVECs were purchased from the National Collection of Authenticated Cell Culture (Shanghai, China) and maintained in endothelial cell medium (ScienCell, Carlsbad, CA, USA) containing 5% FBS, cell growth supplement, 100 U/ml streptomycins and penicillin in a humidified incubator (ThermoFisher, Waltham, MA, USA) at 37°C with 95% O_2_ plus a 5% CO_2_ atmosphere.

### Matrigel tube formation assay

Matrigel (Corning, Arizona, USA) was coated in 96 well plates at 4°C for 30 min and then was polymerized at 37°C for 30 min. HUVECs were seeded in matrigel at seeding density of 2 × 10^4^ and incubated in endothelial cells medium containing 2% FBS. HUVECs were treated with 20 μM DCPIB (Tocris Bioscience, Bristol, UK) and DMSO (Sigma-Aldrich, MO, USA) in the presence or absence of VEGF165 (Catalog.48143, CST, Danvers, Massachusetts, USA) for 48 h and were then photographed using an inverted phase contrast microscope (Olympus, Tokyo, Japan). HUVECs (passage 6–8) were used for *in vitro* experiments.

### Cell proliferation assay

HUVECs seeded in 6 well plates were incubated until about 70–80% confluence followed by 8–12 h of starvation. After starvation, cells were treated with DCPIB and DMSO in the presence or absence of VEGF165 for 48 h. Cell proliferation assay was assessed by Edu cell proliferation kit (Ribobio, Guangdong, China) based on the combination of Edu and apollo fluorescent dyes to detect newly synthesized DNA.

### Cell migration assay

A scratch wound-healing assay was used for evaluating the effect of DCPIB on cell migration. The HUVECs were seeded in 6-well plates and grown to the confluence at 95%. After 16 h of starvation, pipette tips were used to scratch the monolayers of HUVECs. After being washed twice by PBS, cells were exposed to DCPIB with and without VEGF165 for 24 h. Phase-contrast microscopy was used to take photographs at 0 and 24 h after wounding. The healing rate was measured as fold changes. The distance of the migration was measured using Image J software.

### Immunofluorescence assay

Mice hearts and cornea were embedded in OCT (SAKURA, Torrance, CA, USA). Immunostainings were performed on 7 μm-thick cryosections or on HUVECs on coverslips. 2% PFA was used to fix tissue sections or cells. Primary antibodies and dilutions: CD31 (1:100, ab281583, Abcam, Cambridge, UK), VEGF165 (1:250, ab52917, Abcam, Cambridge, UK) and VEGFR2 (1:500, 9698, CST, Danvers, MA, USA). Fluorescently labeled Alex 594 secondary antibody (1:200, A32754, A48278, Invitrogen, Waltham, MA, USA) were used. DAPI visualized cell nucleus. Fluorescent signals were detected with a confocal laser scanning microscope (LSM980, Zeiss, Oberkochen, Germany).

### Western blotting

RIPA lysis buffer supplemented with phosphatase and proteinase inhibitor (Roche, Basel, Switzerland) was used for protein extraction. Protein from cell lysates or tissues was separated on SDS-PAGE gel and transferred onto PVDF membrane (Merck Millipore, # IPVH00010). Western blot analysis was then performed using primary antibodies against VEGF165 (1:1,000, CST, #50661), VEGFR2 (1:1,000, CST, #9698), phospho-VEGFR2 (1:1,000, Tyr1175, CST, #2478), phospho-STAT3 (1:1,000, Tyr705, Abcam, #ab267373), STAT3 (1:2,000, Abcam, #ab68153), ERK (1:3,000, Abcam, #ab184699), phospho-ERK (1:1,000, Thr202/204, CST, #4370), RhoA (1:5,000, Abcam, #ab187027), phosphor-AKT (1:1,000, ser473, CST, #4060), AKT (1:1,000, CST, #9272), Cyclin D1(1:200, Abcam, #ab16663) and RhoA (1:5,000, Abcam, #ab187027). Secondary antibodies were employed to visualize the immunoblots. Image J software was utilized to quantitatively analyze protein expression.

### MTT assay

MTT assay was used to measure cell viability (Sigma-Aldrich, #11465007001) as described previously (21) with minor modifications. HUVECs were treated with various concentrations of DCPIB (5, 10, 20 and 50 μM) for 24 h. 10 μl MTT labeling solution was added to each well for a desired final concentration of 0.5 mg/ml. After 4 h of incubation, medium was aspirated and then washed with PBS. 200 μl DMSO per well was added and placed in a shaker for dissolving the dye. The cultures were solubilized and the absorbance value was measured at a wavelength of 570 nm with a universal microplate reader (ELX800, BioTek Instruments Inc, Vermont, USA).

### Drug toxicity assessment *in vivo*

Survival curves and liver function were used for assessing the DCPIB toxicity *in vivo*. Survival curves were obtained using the Kaplan-Meier estimator with mice treated with DCPIB and DMSO. Liver function index including ALT (G Biosciences, #IT5508) and AST (Elabscience, #E-EL-M0160) was observed by mouse ELISA kit.

## Results

### DCPIB suppresses angiogenesis in the corneal suture (CS) model and myocardial infarction (MI) model

To determine the role of DCPIB (Supplementary Figure 1A) in angiogenesis *in vivo*, CS and MI models were used. In the first step, we tested the potential toxicity of DCPIB on mice under basal conditions *in vivo*. We found that intraperitoneal administration of DCPIB at 15 mg/kg/days in mice was reasonable and safe due to no evidence of death and liver injury (Supplementary Figures 1B–D).

**Figure 1:**
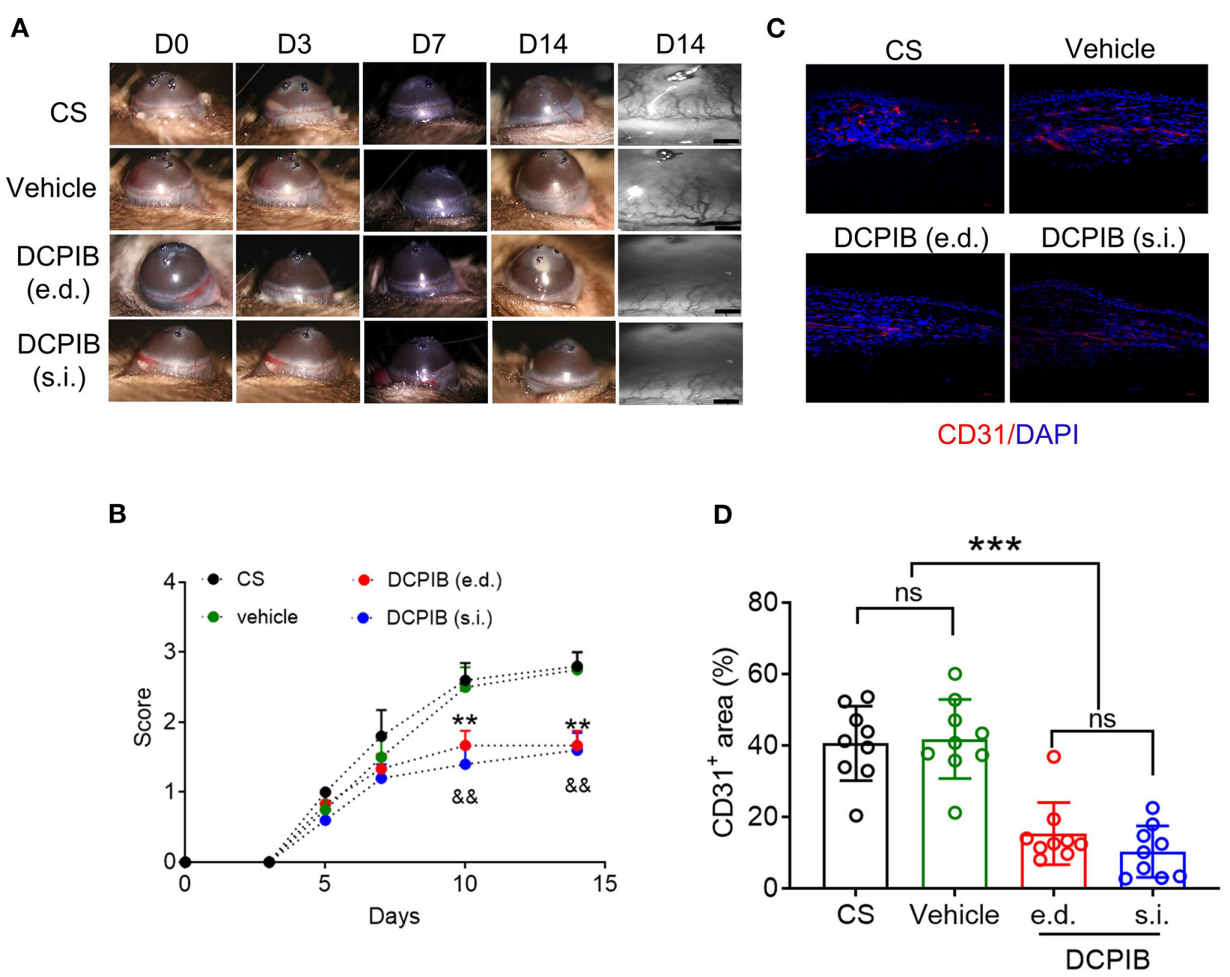
DCPIB reduced mouse corneal neovascularization. **(A,B)** Representative slit-lamp images and semi-quantitative analysis of corneal neovascularization at days 0, 3, 5, 7, 10 and 14 after corneal suture (CS) in the presence or absence of DCPIB treatment with eye drop (e.d.) and subconjunctival injection (s.i.) (*n* = 5~6). **(C,D)** Representative immunofluorescence and quantitative analysis of the CD31 level in the cornea of CS mice treated with DCPIB or vehicle for 14 days (*n* = 9). Red fluorescence represents CD31 and blue fluorescence represents the nuclei (stained by DAPI). Scale bar = 50 μm. **p < 0.01 vs. CS, ^&&^*p* < 0.01 vs. CS, ***p < 0.001, *ns*, no significance.

In the CS model, slit-lamp biomicroscopy was used for observing corneal neovascularization in mice treated with DCPIB on days 0, 3, 5, 7, 10, and 14, and the acquired images were analyzed using a semi-quantitative corneal neovascularization score. We found that subconjunctival injection of 10 μM DCPIB and eye drop of 300 μM DCPIB both markedly inhibited corneal neovascularization on days 10 and 14 after the procedure (Figures 1A,B). Immunofluorescence staining of corneal tissue showed CD31 positive ECs in the CS group and vehicle group, while CD31 expression was significantly decreased after DCPIB treatment (Figures 1C,D).

**Figure 2:**
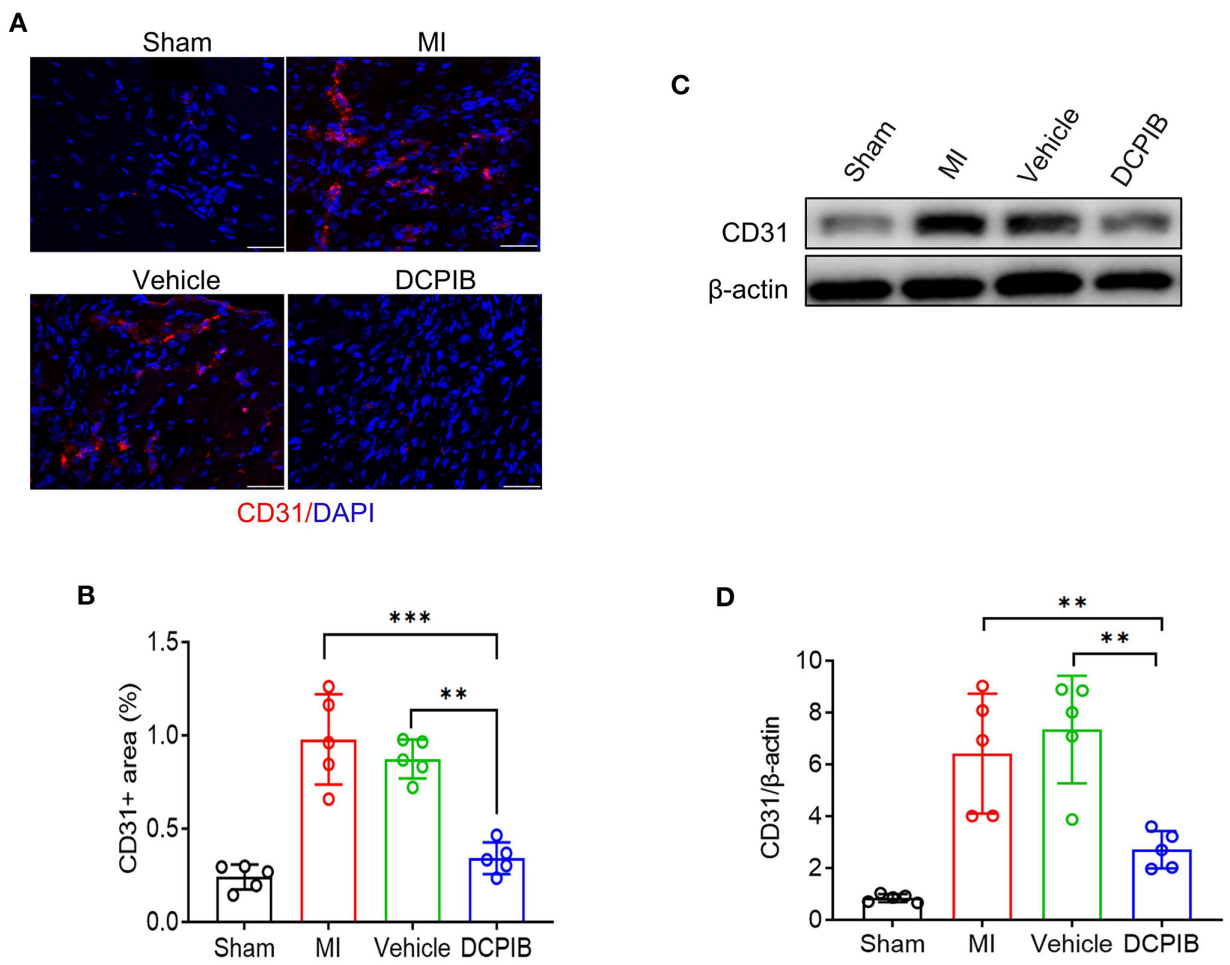
DCPIB inhibited angiogenesis after MI. **(A,B)** Representative immunofluorescence and quantitative analysis of the CD31 level in the infarct border zone of the heart tissue of mice treated with DCPIB for 14 days after MI (*n* = 5). Red fluorescence represents CD31 and blue fluorescence represents the nuclei (stained by DAPI). Scale bar = 50 μm. **(C,D)** Representative western blot and quantitative analysis of CD31 protein level in the heart tissue of MI mice infused with 15 mg/kg/day DCPIB or vehicle for 14 days (*n* = 5). **p < 0.01, ***p < 0.001, *ns*, no significance.

The inhibitory effect of DCPIB on angiogenesis was further supported in the MI model. Herein, Mice were *ip* administered DCPIB (15 mg/kg/days) for ~21 days (7 days before and 14 days after the procedure). Echocardiography was performed on days 7 and 14 after the procedure. No significant changes in cardiac function were found in DCPIB-treated mice, in comparison with the vehicle group and MI group (Supplementary Figure 3). Of note, we found that CD31 positive ECs and protein expression in DCPIB-treated mice was lower than MI and vehicle group (Figure 2). These results suggested that DCPIB exerted an anti-angiogenesis effect in the injury- and ischemia-induced angiogenesis models.

### DCPIB inhibits tube formation, proliferation and migration in HUVECs

Angiogenesis is closely associated with ECs proliferation, tube formation and migration (22). Therefore, we further investigated whether DCPIB would also repress HUVECs tube formation, proliferation and migration.

**Figure 3:**
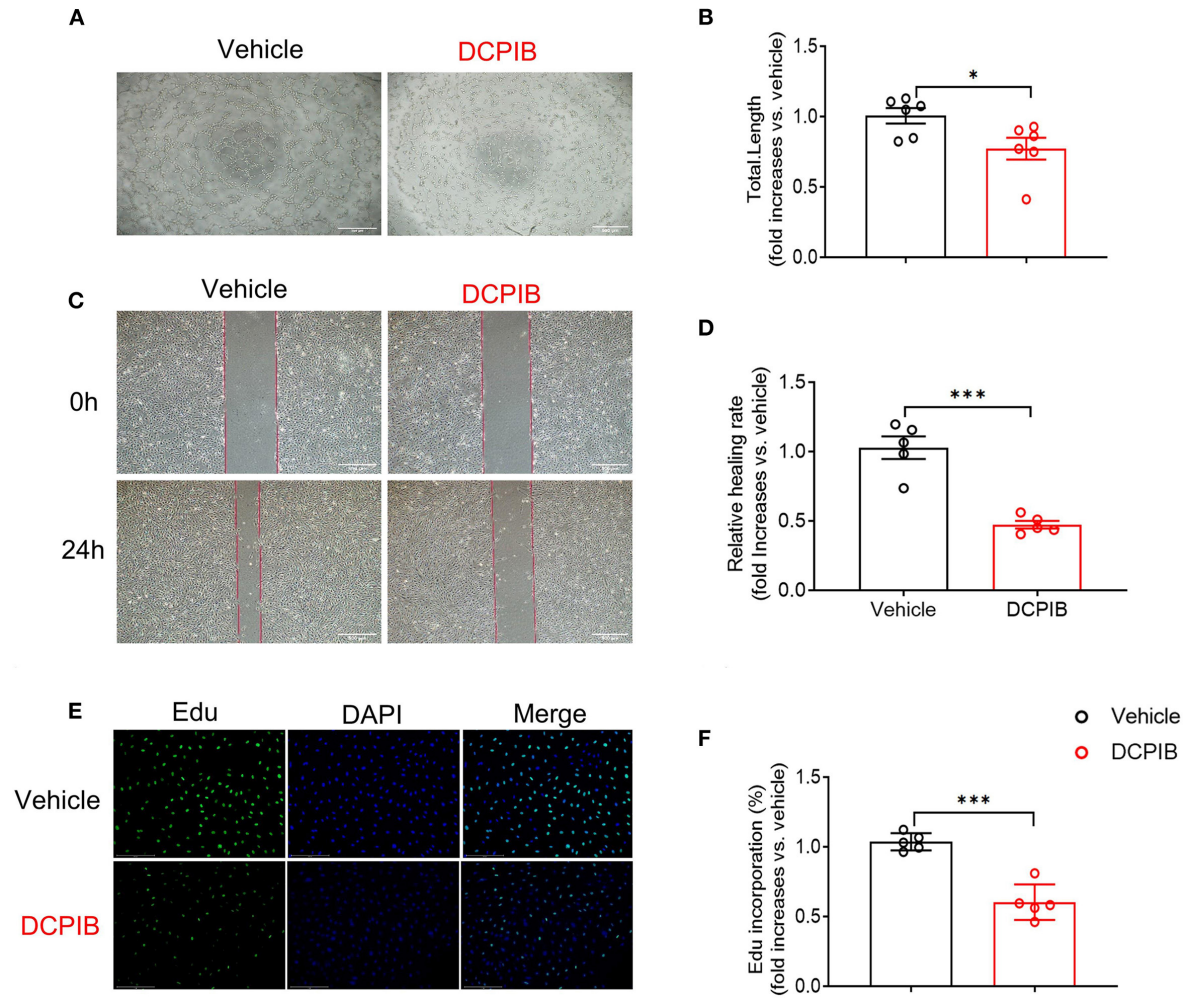
DCPIB inhibited angiogenesis *in vitro*. **(A,B)** Images and quantitative results of spontaneous tube formation of HUVECs with or without 20 μM DCPIB (*n* = 6). Scale bar = 500 μm. **(C,D)** Images and quantification of HUVECs wound healing assays in the presence or absence of DCPIB (*n* = 5). Scale bar = 500 μm. **(E,F)** Images and quantitation of proliferative ability of HUVECs with or without DCPIB evaluated by Edu staining (*n* = 5). Edu (green), DAPI (blue). Scale bar = 100 μm. *p < 0.05, ***p < 0.001.

**Figure 4:**
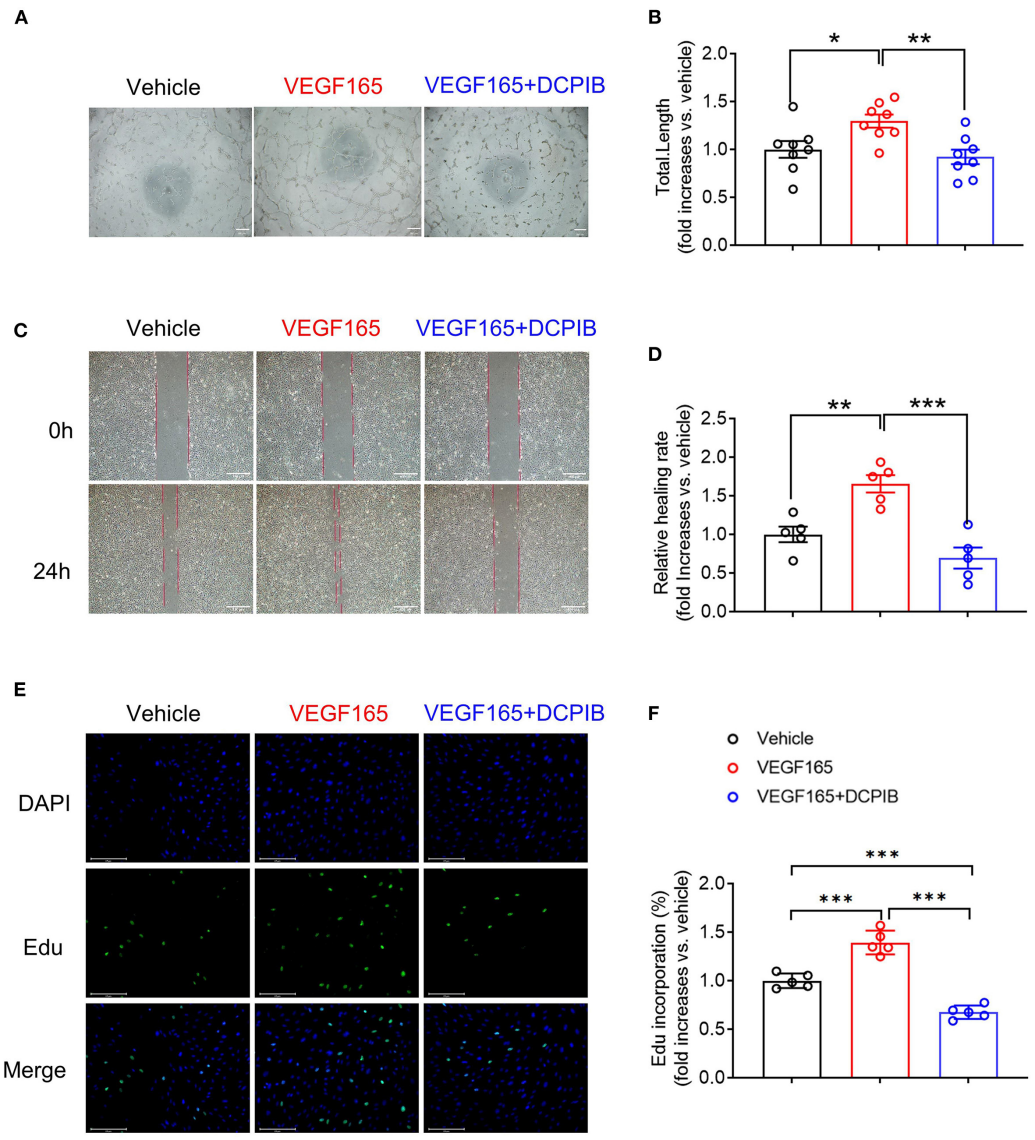
DCPIB inhibited VEGF165-induced angiogenesis *in vitro*. **(A,B)** Representative images and quantitation of VEGF165 induced HUVECs tube formation in the presence or absence of 20 μM DCPIB (*n* = 8). **(C,D)** Representative images and quantitation of VEGF165 induced HUVECs migration assessed by wound healing assay in the presence or absence of DCPIB (*n* = 5). **(E,F)** Representative images and quantitation of VEGF165 induced HUVECs proliferation evaluated by Edu staining in the presence or absence of DCPIB (*n* = 5). Edu (Green), DAPI (blue). Scale bar = 100 μm. *p < 0.05, **p < 0.01, ***p < 0.001.

MTT assay was performed for observing the effect of DCPIB on cell viability. We found that DCPIB (5, 10 and 20 μM) did not affect cell viability (Supplementary Figure 2). A high concentration of 20 μM DCPIB was used in *ex vivo* experiments. Briefly, at 24 h after adding DCPIB, the significant reduction in the formation of HUVECs into the capillary-like network and the total tubule length under basal and VEGF165-induced conditions on matrigel was observed (Figures 3A,B, 4A,B). In addition, migration was assessed by wound-healing assay. HUVECs migration was effectively inhibited by DCPIB in the presence or absence of VEGF165 (Figures 3C,D, 4C,D). Moreover, Edu staining assay was employed to investigate the proliferation rates of HUVECs treated with DCPIB in the presence or absence of VEGF165. The data showed that HUVECs proliferation was decreased in the cells treated with DCPIB in the presence or absence of VEGF165 (Figures 3E,F, 4E,F). Collectively, these data indicate that DCPIB inhibited HUVECs tube formation, migration and proliferation *in vitro*, which are required for angiogenesis.

### DCPIB modulates the VEGFR2 signaling pathway in HUVECs

**Figure 5:**
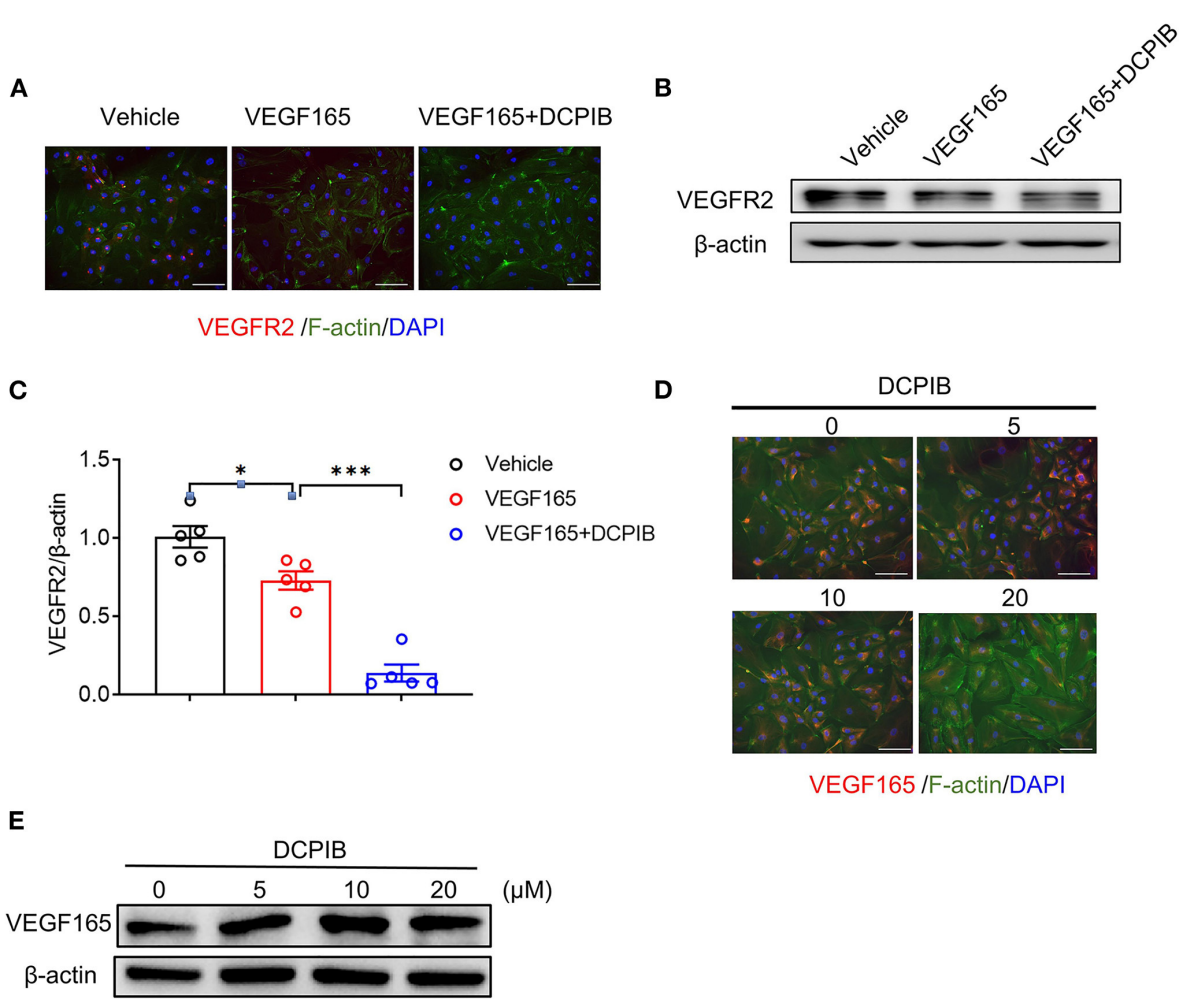
DCPIB reduced VEGFR2 expression. **(A)** Representative images of Immunofluorescence of VEGFR2 expression in HUVECs treated with vehicle, VEGF165 and DCPIB for 48 h (*n* = 3). VEGFR2 (red), DAPI (blue) and F-actin (green). Scale bar = 50 μm. **(B,C)** Representative western blot and quantitative analysis of the VEGFR2 protein expression in HUVECs treated with vehicle, VEGF165 and DCPIB for 48 h (*n* = 5). **(D)** Immunofluorescence detection of VEGF165 expression in HUVECs treated with DCPIB (0, 5, 10, 20 μM). VEGFR2 (red), DAPI (blue) and F-actin (green). Scale bar = 50 μm. **(E)** Western blot analysis of VEGF165 expression in HUVECs treated with DCPIB (0, 5, 10, 20 μM). *p < 0.05, ***p < 0.001.

**Figure 6:**
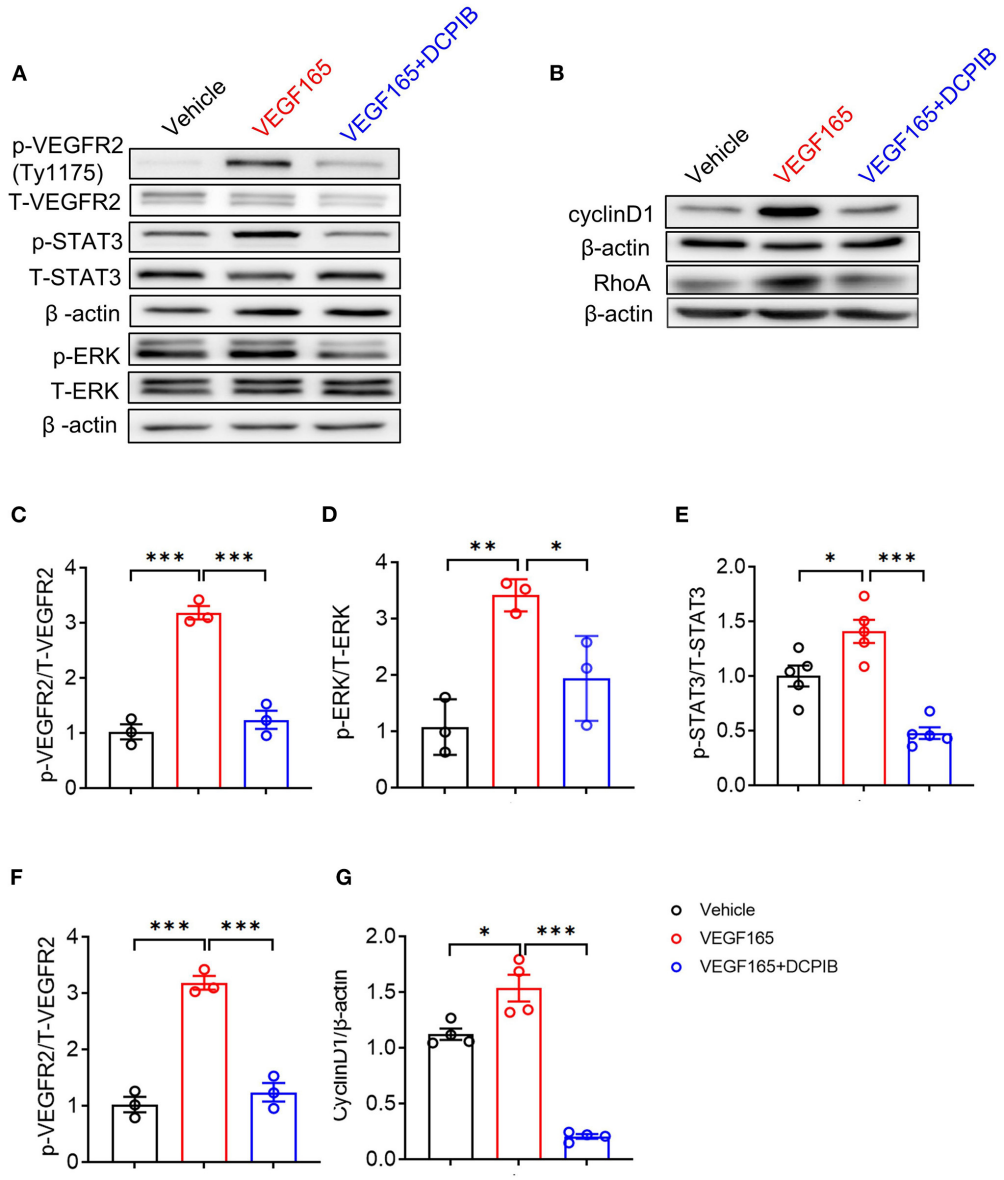
DCPIB inhibited VEGFR2 phosphorylation and its downstream signaling pathways. **(A)** Western blot analysis of p-VEGFR2 (Tyr1175), p-STAT3 and p-ERK levels in HUVECs treated with vehicle, VEGF165 and DCPIB for 10 min. Total VEGFR2, STAT3, ERK and β-actin were used as the loading control (*n* = 3~5). **(B)** Western blot analysis of RhoA and Cyclin D1 protein expression in HUVECs treated with vehicle, VEGF165 and DCPIB for 48 h. β-actin was used as the loading control (*n* = 4~5). **(C–G)** Quantitative analysis of p-VEGFR2, p-STAT3, p-ERK, RhoA and Cyclin D1 protein expression. *p < 0.05, **p < 0.01, ***p < 0.001.

The VEGF/VEGFR2 signaling pathway is the major regulator of angiogenesis in the endothelium (23). We next investigated whether DCPIB might affect the VEGF–VEGFR2 signaling pathway in HUVECs. We found that increasing the concentration of DCPIB (5~20 μM) did not alter VEGF165 protein expression (Figures 5D,E). Upon VEGF165 stimulation, a reduction in the phosphorylation (Figures 6A,C) and total (Figures 5A–C) of VEGFR2 were detected in the cells treated with DCPIB. Thus, we speculated that DCPIB would affect VEGFR2 downstream signaling. Indeed, phosphorylation of STAT3 and ERK in response to VEGF165 was decreased in the cells treated with DCPIB for 10 min (Figures 6A,D,E). Total RhoA and cyclin D1 protein levels were markedly reduced in the cells treated with DCPIB for 48 h (Figures 6B,F,G). These data indicated that DCPIB negatively regulates VEGFR2 and downstream signaling proteins.

### Computational modeling of the interaction between DCPIB and VEGFR2

**Figure 7:**
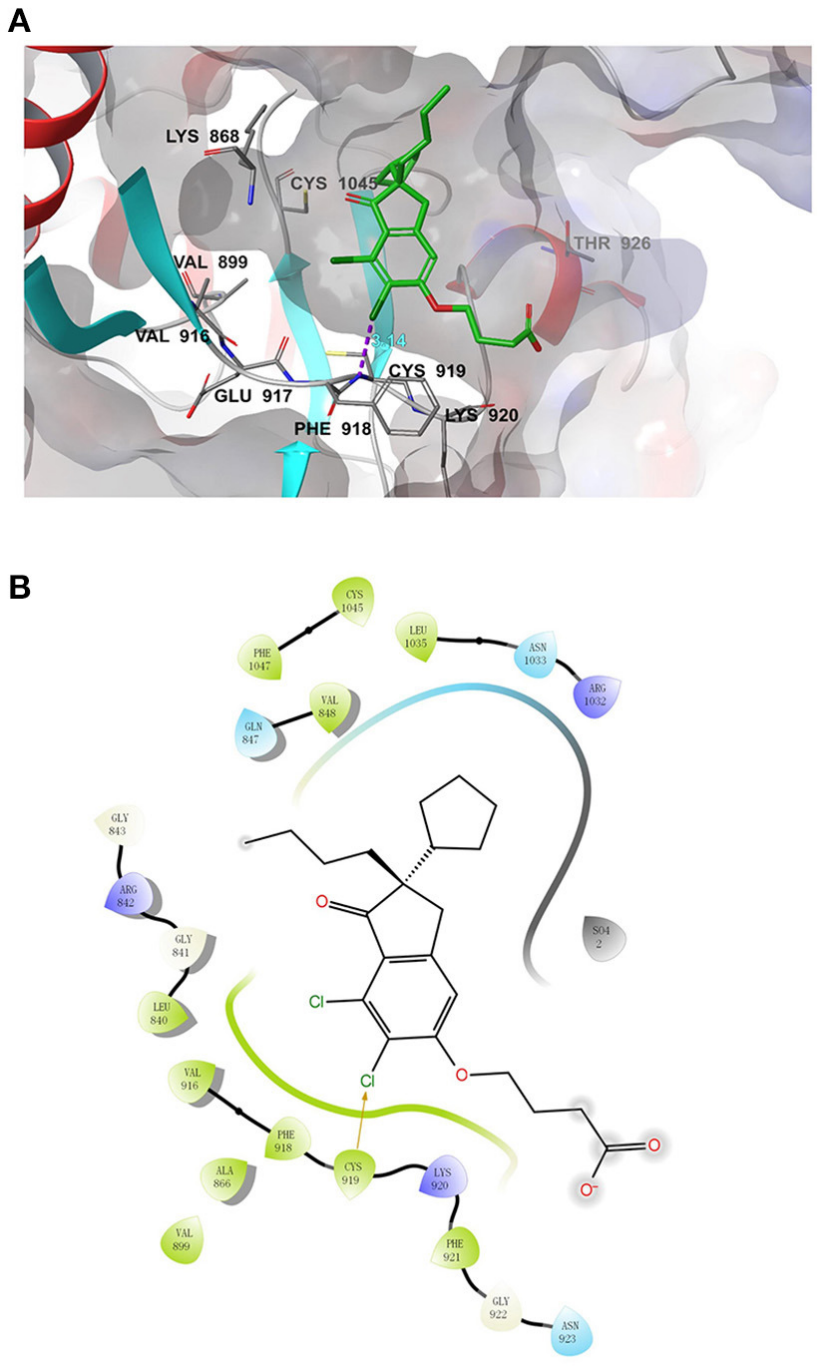
Molecular docking simulation analysis of DCPIB. **(A)** Molecular modeling of the interaction between DCPIB and VEGFR2. Docking pose of VEGFR2 with DCPIB was shown in the graph. **(B)** Docking pose of DCPIB with VEGFR2 using a two-dimensional diagram.

We further performed a molecular docking simulation to analyze whether DCPIB binds to VEGFR2. Molecular docking of Nintedanib (positive control) to VEGFR2 revealed the “ligand-binding pocket” of VEGFR2 (Supplementary Figure 4). Table 1 shows the docking score and binding energy of Nintedanib and DCPIB with VEGFR2. The docking pose of DCPIB has chloride atom interactions with Cys 919 residues of VEGFR2 and form a stable docking pose (Figure 7A). Figure 7B further demonstrated the two-dimensional ligand-receptor interaction diagram between DCPIB and VEGFR2. Taken together, computational modeling further supported that DCPIB may bind to VEGFR2 to disrupt the VEGF/VEGFR2 signaling pathway.

**Table 1:** Docking result of DCPIB and VEGFR2.

**Compound**	**MMGBSA binding energy (kcal/mol)**	**Docking score (kcal/mol)**
Nintedanib	−72.31	−9.498
DCPIB	−34.55	−5.520

### Statistical analysis

Continuous variables in the present study are present as mean ± SEM. Statistical comparisons were assessed with Student's *T*-test for two groups, and with one-way ANOVA followed by the Student- Newman-Keuls for multiple groups. GraphPad Prism Software (9.0 version) was utilized for statistical analysis. A *p*-value < 0.05 was considered significant.

## Discussion

Angiogenesis is essential for tissue repair and development. Abnormal angiogenesis, however, is a crucial pathogenic factor of corneal injury and tumor progression. Angiogenesis is a double-edged sword. In the case of ophthalmology angiogenesis and cancer, angiogenic inhibitors can be used to reduce angiogenesis. In 2014, Visudyne was the first FDA-approved anti-angiogenic therapy for eye disease. Bevacizumab, sorafenib and pazopanib have been approved by FDA to treat cancer *via* the inhibition of angiogenesis. In the case of ischemic diseases, such as ischemic stroke and ischemic heart disease, however, proangiogenic agents can be used to induce therapeutic angiogenesis. In a clinical trial, the administration of bFGF protein in severe coronary artery disease patients, who were not suitable for coronary artery bypass grafting and percutaneous coronary intervention, was feasible and safe. All in all, angiogenesis-based therapy is a promising strategy for certain human diseases. There is an urgent need to discover new compounds targeting angiogenesis.

DCPIB was originally identified as a highly selective and potent antagonist of VRAC and widely applied in exploring the physiological and pathological function of VRAC (24). The inhibitory effect of DCPIB on VRAC is voltage-independent (7). Several lines of evidence found that DCPIB was capable of additive protection against the reversible middle cerebral artery occlusion model and common carotid artery occlusion model *via* inhibition of VRAC (25, 26). The neuroprotective effects of DCPIB were responsible for the suppression of glutamate release *via* VRAC (26). In addition, DCPIB modulated the action potential of atrial cardiomyocytes, suggesting that DCPIB is a valuable compound to study the chloride current in cardiac excitability (7). DCPIB restored glycemic control, reduced hepatic steatosis and improved insulin sensitivity in diabetic mice independently of VRAC (17). Of note, several lines of evidence found that VRAC participated in the regulation of angiogenesis by affecting ECs proliferation and cell cycle (27). VRAC blockers including NBBP, mibefradil, tamoxifen and clomiphene inhibited tube formation (13). In addition, DCPIB belongs to the class of oxobutyric acid which is an oxo derivative of butyric acid. A previous study revealed the antiangiogenic role of butyric acid *via* inhibition of VEGF/KDR gene expression and endothelial cell proliferation (28). Thus, we speculate that DCPIB serves as angiogenesis inhibitor.

In the present study, we employed angiogenesis models including CS and MI to verify our speculation and provided evidence for the first time supporting the anti-angiogenesis role of DCPIB in angiogenesis. This finding is consistent with the effect of VRAC blockers on angiogenesis (13). In addition, endothelial-specific knockout LRRC8A, the key component of VRAC, resulted in the inhibition of growth of blood vessels and reduction in microvascular density (15), indicating that VRAC positively regulated angiogenesis. Intriguingly, DCPIB enhanced vascularity in hind limb ischemia *via* reduced intracellular chloride concentration (16); but in fact, there appeared to be no solid evidence to support the conclusion due to the lack of measurement of chloride concentration *in vivo*. However, a recent study found that low-dose, long-term DCPIB administration improved systemic metabolism in type 2 diabetes *via* upregulation of LRRC8A in adipocytes (17). Taken together, the effect of DCPIB on VRAC/LRRC8A mainly depends on dosage, cell type and other factors, which might account for the “contradictory effect” of DCPIB on angiogenesis.

ECs migration, proliferation and tube formation are essential steps for angiogenesis. We next examine the effect of DCPIB on ECs migration, proliferation and tube formation *in vitro*. We found that DCPIB inhibited spontaneous and VEGF-induced HUVECs migration, proliferation and tube formation. Targeting angiogenesis has largely focused on VEGF/VEGFR2 signaling due to its crucial role in angiogenesis (29). VEGF165 is a major stimulator of angiogenesis and is widely expressed in a variety of human and animal tissues. VEGF165, secreted by ECs and other tissues, promotes ECs proliferation, migration and vessel formation (30). In addition, VEGF165 is a key inductor of vascular permeability due to its pro-inflammatory properties (31). In the preclinical study, blockade of the VEGF165 pathway led to a reduction in infarct size (32). MI share common pathophysiology with cerebral ischemia. Conteracting the VEGF pathway reduced cerebral edema and tissue injury after ischemia/reperfusion injury in mice (33). In this study, we found that DCPIB did not reduce endothelial VEGF165 expression *in vitro* and affect cardiac function after MI.

VEGF plays its role by binding to VEGF receptors on the ECs and subsequently activating the downstream signaling pathway related to angiogenesis. VEGFR2 is the major receptor of VEGF. VEGF/VEGFR2 signaling pathway is crucial for ECs survival, migration, proliferation and tube formation. VEGFR2 modulates HUVECs survival by activation of the Src/PI3K/AKT signaling pathway (34) and then AKT directly phosphorylates apoptosis proteins for ensuring cell survival. VEGFR2 is required for mediating ECs proliferation in angiogenesis *via* the PLCγ/PKC/ERK signaling pathway. VEGF activated PLCγ by phosphorylation of the C-terminal Y1175 of VEGFR2. Activation of PLCγ hydrolyzed PIP2 and produced DAG that activates PKC, which promoted translocation of ERK to the nucleus and regulates transcription activity in response to extracellular stimuli (23). The endothelial AKT/ERK/CyclinD1 signaling pathway participates in angiogenesis (35). A variety of intracellular signaling pathways modulated by VEGFR2 are involved in ECs migration. VEGF-induced phosphorylation of VEGFR2 (Y1175) bound the Src homology domain 2 of SHB, and then prevented PI3K activation and migration (36, 37). The phosphorylation of VEGFR2 (Y951) bound T cell-specific adapter in ECs, interacted with Src to form a signaling complex, and subsequently modulated HUVECs migration (38). Another phosphorylated site Y1214 of VEGFR2 was implicated in the VEGFR2-mediated cell migration (39). Chronic stress drove angiogenesis *via* the activation of the VEGFR2/STAT3 signaling pathway (40). We, therefore, examined the effect of DCPIB on VEGFR2 and found that DCPIB significantly decreased the total and phosphorylated VEGFR2 in HUVECs. Moreover, DCPIB reduced the expression of VEGFR2 downstream signaling proteins, such as p-STAT3, p-ERK, RhoA and Cyclin D1. In addition, many studies showed that NRP1 is known as a strong modulator of angiogenesis through binding to VEGF and VEGFR2 (41). However, our results revealed that DCPIB did not influence mRNA expression of NRP1 in HUVEC and the predictive docking score of DCPIB-NRP1 was poor (data not shown).

It's important to efficiently design and evaluate novel compounds targeting angiogenesis for pro-and anti-angiogenic therapy. Based on the studies showing the relationship between VRAC/LRRC8A and angiogenesis, we utilized a cell-based model to evaluate the effect of DCPIB, the most potent and specific VRAC blocker, on angiogenesis. Targeted VEGFR2 antagonist and/or agonist designed to interfere with abnormal angiogenesis remains a major focus in the drug development for the treatment of diseases related to angiogenesis. Recently, emerging evidence demonstrated that structure-based *in silico* technology might provide strong support for rapidly identifying the potential and promised compound (1). We found in this study that the docking pose of DCPIB has chloride atom interactions with Cys 919 residues of VEGFR2 and form a stable docking pose. Furthermore, utilizing the structure-based approaches to identify and evaluate additional novel targeting VEGFR2-based compounds may provide promising candidates for angiogenesis-based treatment.

**Figure 8:**
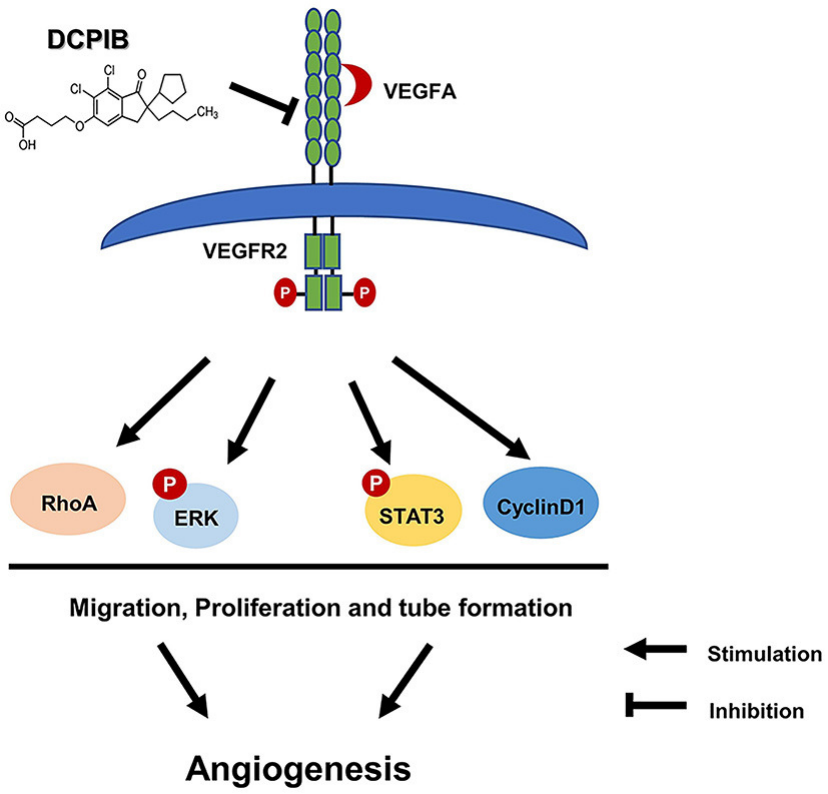
Schematic diagram of effects of DCPIB on angiogenesis. DCPIB exerted anti-angiogenic effect on HUVECs by inhibiting the activation and expression of VEGFR2 and its downstream mediators including RhoA, CyclinD1, p-ERK and p-STAT3, which lead to decreased migration, proliferation and tube formation in endothelial cells.

In the present study, we report the antiangiogenic role of DCPIB in injury- and ischemia-induced angiogenesis mice models. In line with this finding, DCPIB significantly inhibited HUVECs tube formation, proliferation and migration *in vitro* through the inhibition of VEGFR2 expression and phosphorylation. Furthermore, we observed that DCPIB reduced activation and expression of downstream signaling pathways associated with VEGFR2, including p-ERK, p-STAT3, Cyclin D1 and RhoA (Figure 8). To our knowledge, this is the first report that DCPIB suppresses angiogenesis *via* disrupting VEGFR2 signaling pathway. These results support the potential of DCPIB as a promising lead compound in developing anti-angiogenesis drugs in future.

## Data availability statement

The data presented in the study are deposited in the “jianguoyun” repository, accession number https://www.jianguoyun.com/p/DSWGHjwQuITbChjv19gEIAA.

## Ethics statement

The animal study was reviewed and approved by the Animal Care Committee of Xiamen University.

## Author contributions

TZ, YL, and HZ performed the experiments and analyzed the data. LP, JP, QY, and GL provided technical assistance. LJ, YW, and YZ supervised the study and drafted the manuscript. All authors contributed to the article and approved the submitted version.

## Funding

This work was supported by the grants from National Natural Science Foundation of China (82000463 and 81970283), President's Fund of Xiamen University (20720210103), the Natural Science Fund of Fujian Provincial Department of Science and Technology (2019D019), and the Natural Science Fund of Xiamen Municipal Bureau of Science and Technology (3502Z20214ZD1171, 3502Z20214ZD1175, 3502Z20194072, and 3502Z20209150).

## Conflict of interest

The authors declare that the research was conducted in the absence of any commercial or financial relationships that could be construed as a potential conflict of interest.

## Publisher's note

All claims expressed in this article are solely those of the authors and do not necessarily represent those of their affiliated organizations, or those of the publisher, the editors and the reviewers. Any product that may be evaluated in this article, or claim that may be made by its manufacturer, is not guaranteed or endorsed by the publisher.
