# Supplementary material for: 4-(2-Butyl-6,7-dichloro-2-cyclopentyl-indan-1-on-5-yl) oxobutyric acid inhibits angiogenesis via modulation of vascular endothelial growth factor receptor 2 signaling pathway
Source: Front Cardiovasc Med. 2022 Sep 23;9:969616. doi: 10.3389/fcvm.2022.969616 (PMC9537693; doi:10.3389/fcvm.2022.969616)
Supplement: Supplementary file 1 [file Data_Sheet_1.DOCX]

Supplementary Material


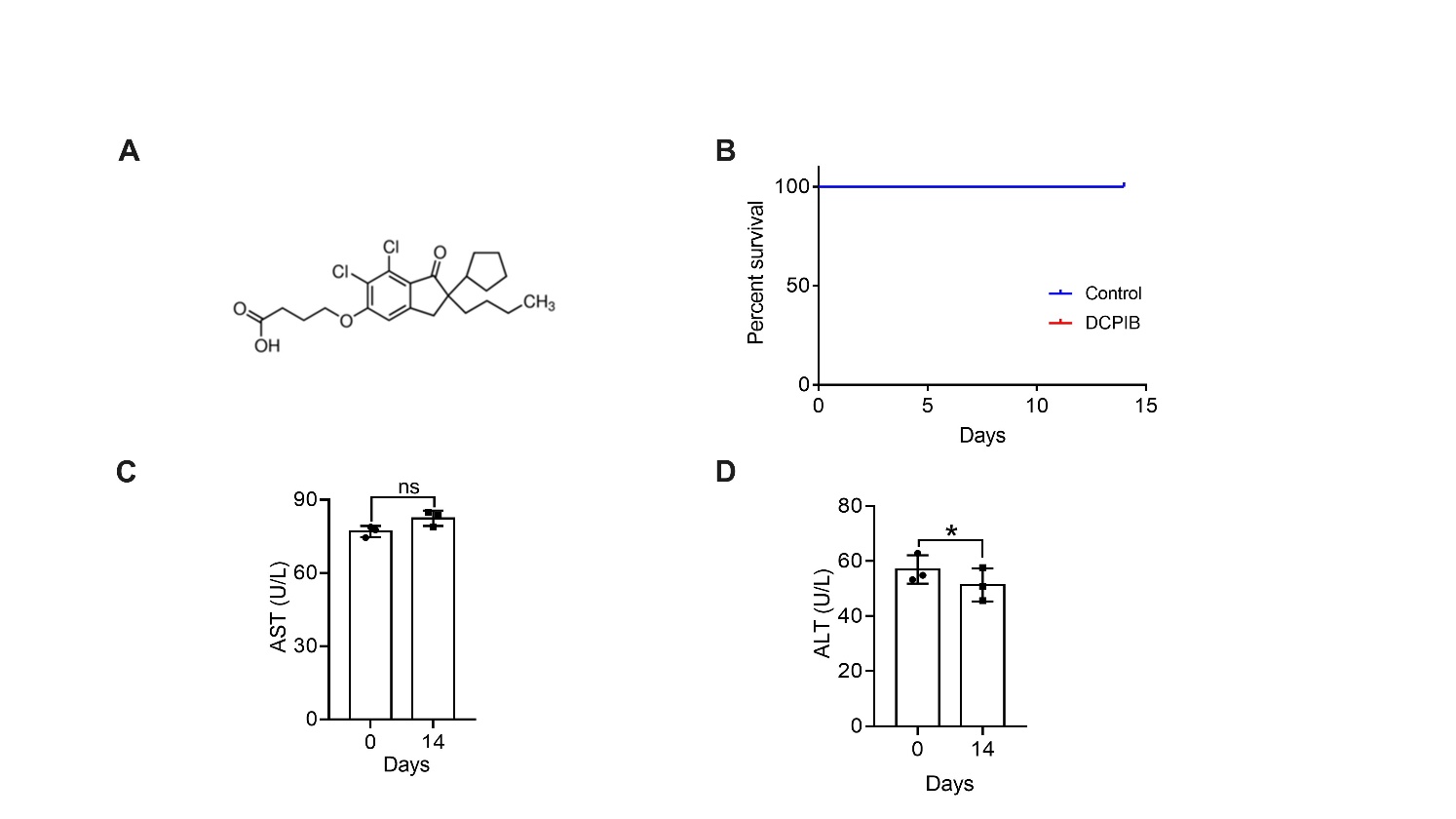


**Supplementary Figure 1.** Evaluation of DCPIB toxicity *in vivo*. (A) DCPIB chemical structure. (B) Overall Survival curves in mice treated with DCPIB (red curve) or Control (blue curve). (C, D) ELISA analysis of serum ALT and AST in mice before and after *ip* treatment of DCPIB (15 mg/kg/day) for 14 days (n=3). ns: no significance; *p＜0.05, ns=no significance.


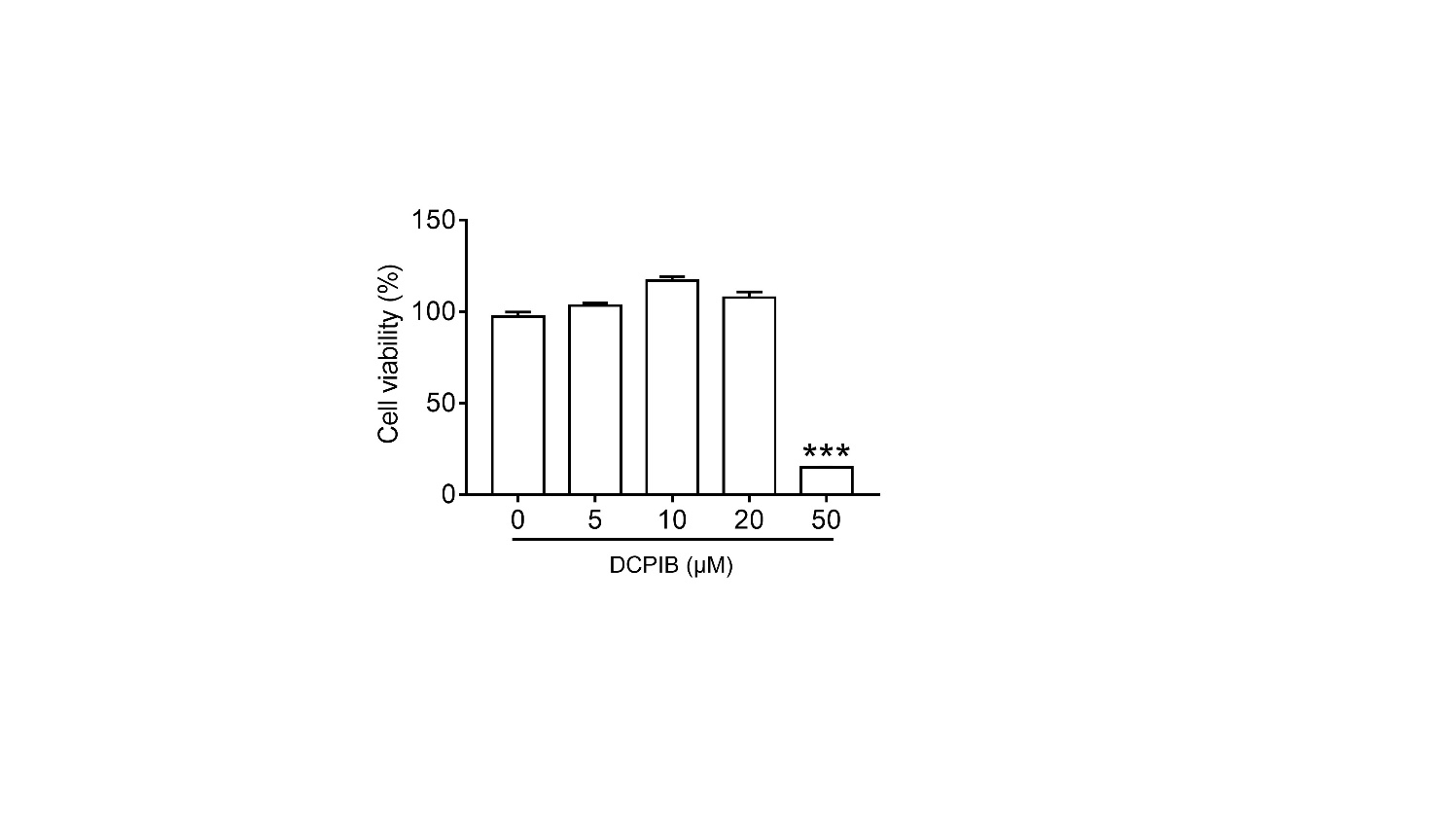


**Supplementary Figure 2.** Effect of DCPIB at different concentration (0, 5, 10, 20, 50 μM) on HUVECs viability (n=8). ***p＜0.001 vs 0 μM group


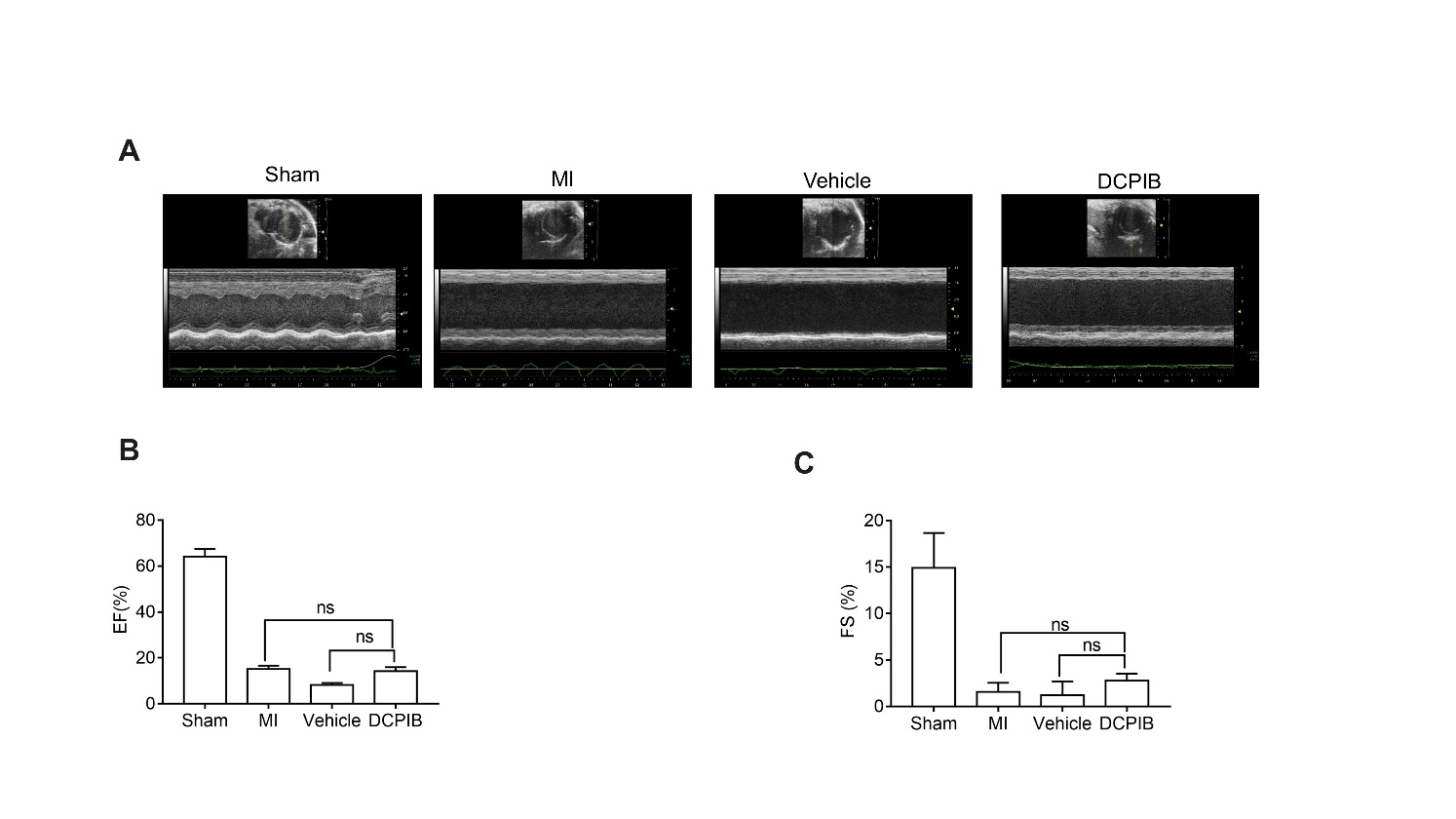


**Supplementary Figure 3.** Effect of DCPIB on cardiac systolic function in mice after MI. (A) Representative M-mode echocardiograms of multiple groups of mice at 14 days after MI. (B, C) Left ventricular ejection fraction (EF) and fractional shortening (FS) measured by echocardiography (n=5~9). ns=no significance.


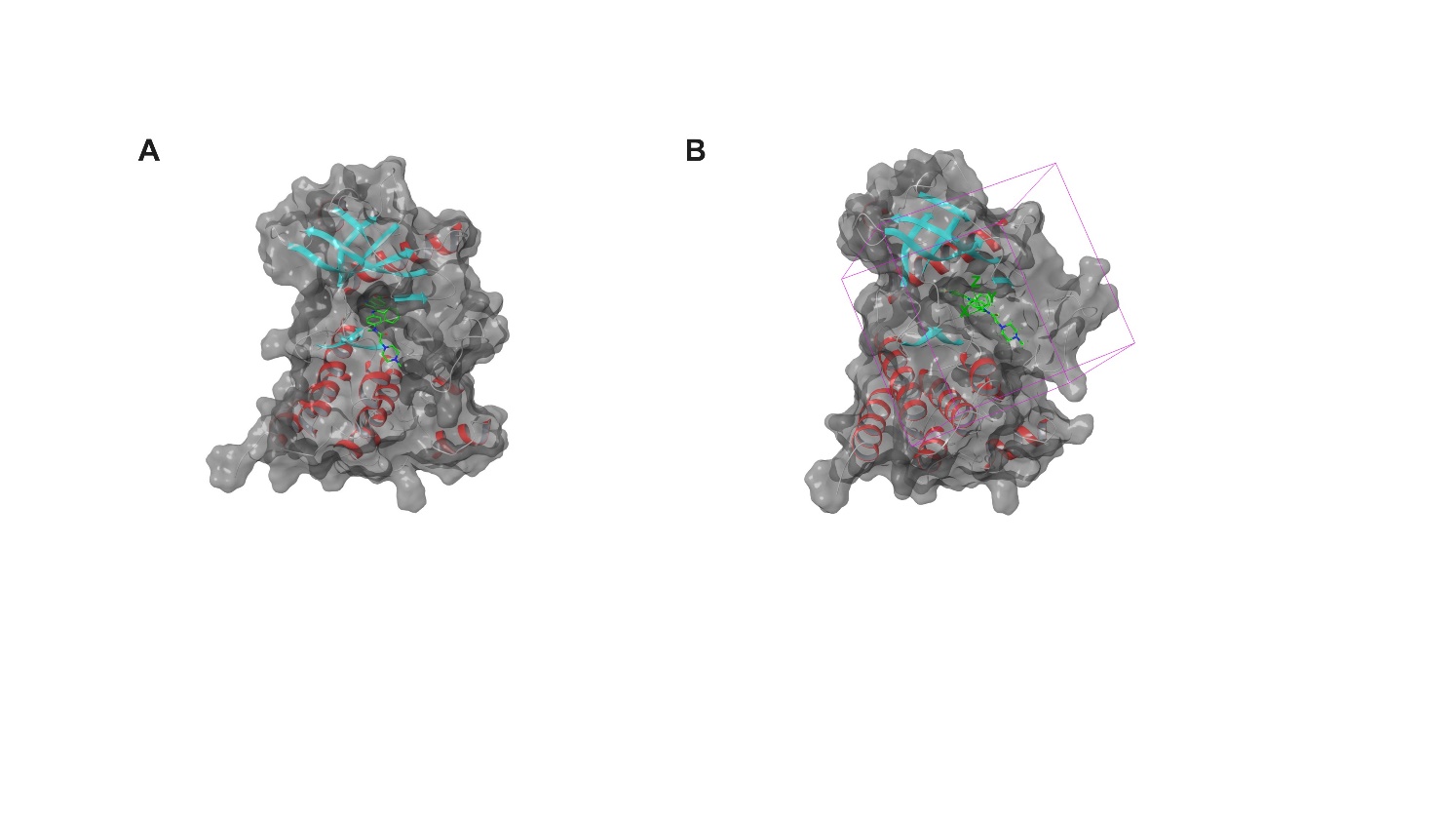


**Supplementary Figure 4.** Three-dimensional structure diagram of ligand binding pocket of VEGFR2
